# Integrated Systems Approach Reveals Sphingolipid Metabolism Pathway Dysregulation in Association with Late-Onset Alzheimer’s Disease

**DOI:** 10.3390/biology7010016

**Published:** 2018-02-09

**Authors:** John Stephen Malamon, Andres Kriete

**Affiliations:** Bossone Research Center, School of Biomedical Engineering, Science and Health Systems, Drexel University, 3141 Chestnut Street, Philadelphia, PA 19104, USA; jsm343@drexel.edu

**Keywords:** Alzheimer’s disease, aging, sphingolipid metabolism, autophagy, co-expression modeling, eQTL, gene set enrichment analysis

## Abstract

Late-onset Alzheimer’s disease (LOAD) and age are significantly correlated such that one-third of Americans beyond 85 years of age are afflicted. We have designed and implemented a pilot study that combines systems biology approaches with traditional next-generation sequencing (NGS) analysis techniques to identify relevant regulatory pathways, infer functional relationships and confirm the dysregulation of these biological pathways in LOAD. Our study design is a most comprehensive systems approach combining co-expression network modeling derived from RNA-seq data, rigorous quality control (QC) standards, functional ontology, and expression quantitative trait loci (eQTL) derived from whole exome (WES) single nucleotide variant (SNV) genotype data. Our initial results reveal several statistically significant, biologically relevant genes involved in sphingolipid metabolism. To validate these findings, we performed a gene set enrichment analysis (GSEA). The GSEA revealed the sphingolipid metabolism pathway and regulation of autophagy in association with LOAD cases. In the execution of this study, we have successfully tested an integrative approach to identify both novel and known LOAD drivers in order to develop a broader and more detailed picture of the highly complex transcriptional and regulatory landscape of age-related dementia.

## 1. Introduction

Alzheimer’s disease (AD) currently afflicts as many as five million Americans and is estimated to reach 14 million by 2050 [1]. Aside from the tremendous health and personal burdens, the current financial burden of AD exceeds $240 billion and is expected to reach nearly $1 trillion by 2050 [2]. Furthermore, there is no current treatment, prevention or cure for AD. It is absolutely imperative that a more comprehensive description of the many complex, systematic dysfunctions involved in AD is aggressively developed with the explicit aim of better equipping the larger scientific community with the tools to tackle this healthcare crisis. Despite myriad confirmed loss-of-function single nucleotide variants (SNVs) that are now associated with LOAD (Late-onset Alzheimer’s disease), there is still no clear understanding of how these variants combine to contribute to the major and irreversible pathological hallmarks of LOAD, such as amyloid plaque deposits, neurofibrillary tangles, and dysfunctions in innate immunity. Whilst many studies focus on rare-variant association and differential expression analysis, here we model co-expression networks, define their function, and test genetic factors that can demonstratively disrupt the homeostasis of a regulatory system or pathway. By combining multiple ‘–omics’ platforms, such as RNA-seq and SNV genotype arrays, we can provide a more robust, detailed description of the many mechanisms that underlie LOAD to gain new insights into the larger, regulatory dysfunctions contributing to this disease.

## 2. Materials and Methods

### 2.1. Workflow Overview

**Figure 1:**
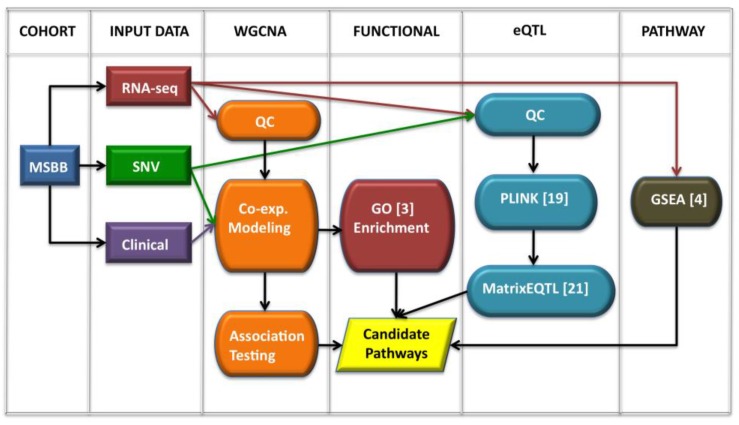
High-level diagram of our integrative analysis workflow: RNA-seq dataset (red) was combined with clinical data (purple) for co-expression modeling and association testing, whereas RNA-seq was combined with SNV genotype data (green) for eQTL analysis. GSEA was used to independently validate candidate pathways.

Our framework, outlined in Figure 1, consists of three main analytical components: co-expression modeling, functional enrichment and expression quantitative trait loci (eQTL) analysis. To assess the validity of this approach, we have designed a pilot study in which we have applied all steps of our workflow to the Mount Sinai Brain Bank’s (MSBB) inferior temporal gyrus dataset (*N* = 58). We used all 58 subjects in our analyses and did not segregate based on disease status, as our sample size was not adequate for a statistically meaningful segregation analysis. Before beginning analysis, we performed a three-tiered quality control process to normalize and reduce the RNA-seq dataset. Co-expression models were constructed using the weighted gene co-expression network analysis (WGCNA) toolkit [3]. Network modules were stored in the topological overlap matrix (TOM), the weighted representation of the Euclidian space of all network-module associations. Next, we performed association-based testing using clinical neuropathological data. From this we identified modules that correlated with specific clinical traits or phenotypes. The TOM was then enriched with functional data provided by the Gene Ontology (GO) consortium [4]. 

We incorporated functional annotation and analysis to elucidate biologically relevant pathways and drive more deeply into the mechanisms underlying LOAD (see Code S1). Next, we used eQTL analysis to examine the relationship between changes in genotype and gene expression. This step allowed us to perform an in silico validation of perturbations in our candidate pathways. This approach gives us the ability to demonstrate how changes in genotype affect larger regulatory systems, not just one gene. Finally, we performed independent, pathway validation using the Broad Institute’s gene set enrichment analysis (GSEA) toolkit [5]. Specifically, we used GSEA to process the RNA-seq data, incorporating the KEGG pathway database [6,7,8], to discover statistically meaningful pathways. By combining these techniques, we have examined how specific genetic variations contribute to the dysregulation of biologically relevant pathways and begin to piece together a story for how these perturbations may lead to systematic failure. 

### 2.2. Data Description

The Mount Sinai Brain Bank (MSBB) Array Tissue Panel study provides RNA-seq, clinical neuropathology and single nucleotide variant (SNV) whole exome sequence (WES) data across 19 brain regions with approximately 60 unrelated, age, and sex-matched samples (~40 LOAD, ~20 controls). For our pilot study, we have chosen to analyse the inferior temporal gyrus (ITG) for two reasons: (1) we determined that it was the highest quality regional dataset and (2) experimental evidence has shown that ITG is associated with early AD pathologies such as memory impairment [9]. 

In this way, we wish to examine early disease conditions with a focus on early intervention and prevention. These subjects range from no cognitive impairment (*N* = 11), mild cognitive impairment (*N* = 18) and severe AD phenotypes (*N* = 29). Table 1 describes the six neuropathology metrics used in this study. Reported race is 45 Caucasian, 10 African, 2 Hispanic and 1 Asian. RNA-seq data were generated using the Affymetrix_133AB or Affymetrix 133Plus2 platforms and were adjusted for the following covariates: pH, post-mortem interval, age at onset, sex and race. WES genotype data were sequenced on the Illumina HiSeq 2500 platform and aligned using the BWA aligner [10] with human reference genome 19 (hg19). All data can be obtained at the following website, provided IRB permission: https://www.synapse.org/#!Synapse:syn5550382.

### 2.3. Analysis of Clinical Data

**Figure 2:**
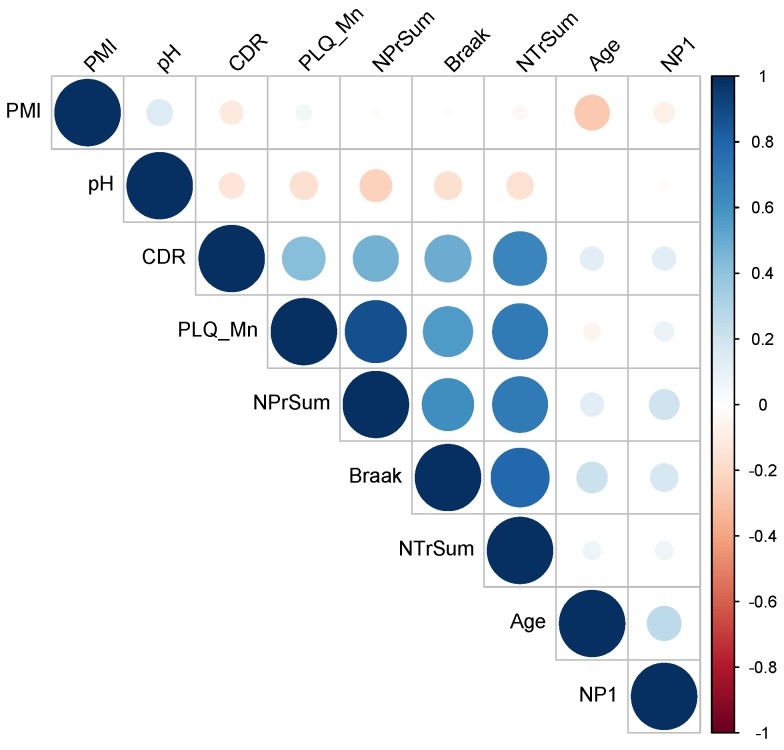
Pearson’s correlation matrix of all nine covariates (post-mortem index, pH, CDR, PLQ_Mn, NPr_Sum, Braak, NTr_Sum, Age, NP1) for all individuals in the MSBB Tissue Array Panel study.

**Figure 3:**
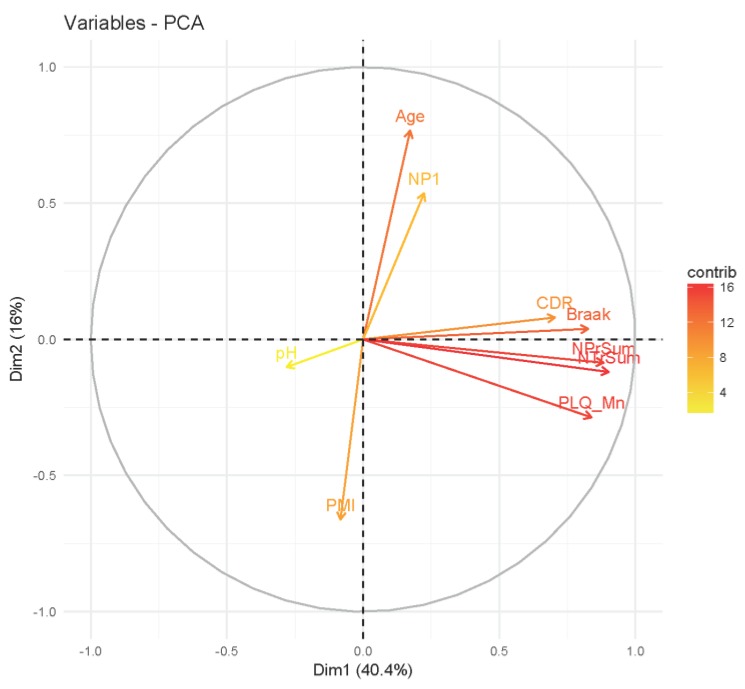
Variables factor map of the 1st and 2nd PCs of clinical neuropathological metrics for all individuals in the MSBB Tissue Array Panel study.

To assess the general concordance of the clinical neuropathology data, we performed Pearson’s correlation (Figure 2) and principal components analysis (PCA) (Figure 3) using FactoMineR [11] on all nine covariates (post-mortem index, pH, CDR, PLQ_Mn, NPr_Sum, Braak, NTr_Sum, Age, NP1, see Table 1 for details). The correlation matrix (Figure 2) shows good concordance with the six main neuropathological metrics. PCA (Figure 3) confirmed a general consensus among the six neuropathological traits with only NP1 deviating in the direction of age. Interestingly, pH moves in the opposite direction of the neuropath data, meaning that pH decreases with respect to disease progression. In conclusion, we feel confident in proceeding with all six metrics in our analysis.

### 2.4. Quality Filtering of RNA-seq Data

**Figure 4:**
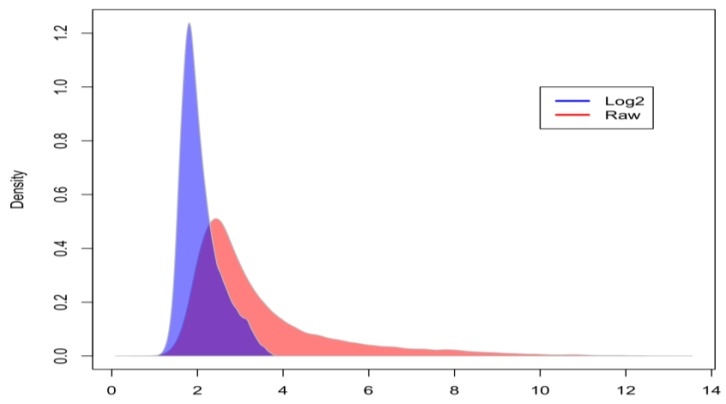
Density plot of RNA-seq data before (red) and after (blue) power transformation (mean = 3.44, SD = 1.701 and mean = 2.069, SD = 0.4604, respectively).

There are many confounding factors when analyzing RNA-seq data. For example, RNA-seq measures transcripts across many different cell populations and neuronal cell types at different stages of pathology and life cycle. To address this inherent heterogeneity, we have developed a comprehensive, three-tiered QC solution to reduce both the noise and number of transcripts analyzed. This included power transformation (PT), entropy, and connectivity filtering. Figure 4 shows the raw RNA-seq (red) data versus the log_2_ transformed data (blue). PT reduced the standard deviation from 1.701 to 0.4604. In addition, we can see that the skewing is significantly reduced after PT. This sequencing platform contains 47,640 probe sets. Our entropy filter leverages the Shannon-Weiner Index of Diversity [12] to measure the informational variability of individual transcripts. We are not interested in transcripts of low variability. We further reduced our dataset by limiting our data to associations above a connectivity threshold as provided by WGCNA. Transcripts with low degrees have fewer connections to other nodes or transcripts and are therefore less likely to have systematic effects on our pathways. By removing transcripts below 3.97 bits and 45 degrees, respectively, we eliminated~27,000 transcripts and continue our analysis with ~20,000 transcripts.

### 2.5. Association Testing

WGCNA [3] allowed for easy network construction (co-expression modeling) and module/pathway identification through hierarchical clustering, so that module eigengenes (1st PC) could be associated with the PCs of clinical traits (2nd PC). Next, we used WGCNA’s network gene screening function (networkScreeningGS [3]) to perform association testing between module eigengenes and clinical principal components, which provided both weighted and standard *p*-values (Fisher’s exact method), Pearson’s correlations, false-discovery rates (FDR) via Benjamini-Hochberg procedure [13], and t-distributions (z-scores) for all network module-associations. Weighted associations were scaled according to the coefficient of the principal component of a given clinical trait.

This allowed us to more accurately assess the relationships between transcriptional networks and their correlation with clinical phenotypes, such as CDR, Braak and mean neuritic plaque density. As a standard quality control procedure, we removed all associations outside of ±3 standard deviations from the mean. Next, we used Bonferroni correction to adjust our *p*-values at a confidence interval (α) of 0.01 with 1,600,000 observations (58 subjects multiplied by 20,000 transcripts). Finally, we enriched our network modules with functional data from the GO consortium, assigning functional terms to each candidate gene.

### 2.6. Expression Quantitative Trait Loci Analysis

**Figure 5:**
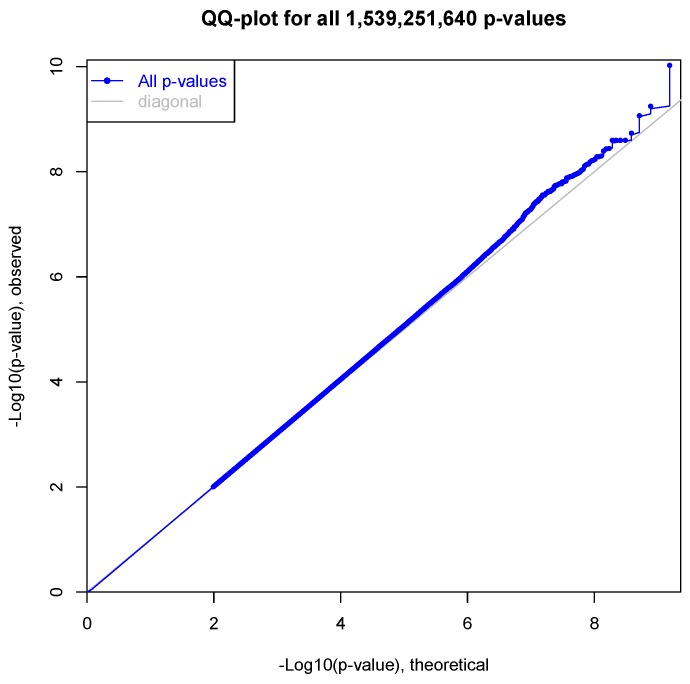
Quantile-Quantile plot of ~1.54 billion *p*-values from eQTL analysis of the MSBB inferior temporal gyrus dataset (*N* = 58).

The last component of our analysis explored potential genetic causes for perturbations in our transcriptional networks. Single nucleotide variation is one such vector. To examine this relationship, we used eQTL analysis to test the relationship between a polymorphism and a change in gene expression. eQTL analysis has been widely used to identify SNVs that lead to changes in gene expression [14,15]. A number of co-expression studies have also been successful in identifying systematic differences in the expression profiles across the mammalian brain to study a number of processes and diseases including LOAD [16,17,18]. Our analytical framework allowed us to combine co-expression modeling with eQTL to closely examine fluctuations that occur in our networks. For our eQTL analysis, we selected the same individuals from the inferior temporal gyrus dataset used in co-expression modeling and association-bases testing. PLINK [19] allowed us to apply variant-level QC procedures and convert genotype files into the standard variant call file (VCF) format. We excluded variants based on a minimum call rate of 0.8, minimum minor allele frequency of 0.02 and a Hardy-Weinberg disequilibrium [20] threshold of 1e^−05^. MatrixEQTL [21] was used to perform linear regression on the genotype and expression datasets with the nine covariates. Over 1.5 billion associations were made (Figure 5) of which 1,048,576 were below the significance threshold of 1e^−05^. Bonferroni correction was applied according to 58 individuals and 34,389 genotypes at a confidence interval of 0.01.

### 2.7. Gene Set Enrichment Analysis

From our association-based results and functional annotation, we observed that genes with the term “sphingolipid” and “myelin” were statistically over-represented in this brain region. In an attempt to directly reproduce this finding, we added a gene set enrichment analysis using the Broad Institute’s GSEA toolkit. GSEA derives weighted, biological enrichment scores using a number of statistical methods. We used the ‘Log2_Ratio_of_Classes’ enrichment factor and tested its association with the KEGG pathway database. For phenotype, we supplied the two classes of cases (*N* = 38) and controls (*N* = 10). 

## 3. Results

### 3.1. Association Testing

Table 2, below, summarizes the gene transcripts that fall below the confidence threshold of 0.05 and contain the term ‘myelin’ or ‘sphingolipid’ in their functional annotation term for this brain region. Please see Table S1 for a full list of associations and terms. GENE is the coding region that represents each transcript. All reported transcripts are of the same species. P.WEIGHTED and COR.WEIGHTED are scaled at 0.5 of the coefficient of the 2nd PC. P.WEIGHTED represents the Bonferroni corrected, lowest observed weighted *p*-value. FDR is calculated using the Benjamini-Hochberg procedure and represents the minimum amount of the Type I error measured in the prediction. COR.WEIGHTED is the Pearson’s correlation of the gene-trait association. #OF TRAITS represents the number of clinical traits observed in this brain region. In summary, we observed a total of 252 transcripts below the significance threshold (0.05) in association with four clinical traits. Of these, 135, 47, 35, and 5 transcripts are significant for PLQ_Mn, Braak, NTr_Sum, and NP1, respectively. Braak and NTr_Sum show good concordance with PLQ_MN at 95.7% and 100%, respectively. Our data shows several genes involved in the sphingolipid metabolic and myelin maintenance pathways. Of note, we also observed previously identified LOAD signals that correlate with changes in gene expression not associated with the aforementioned functional terms, such as GPRC5B [22,23] (*p* = 9.22e^−06^), KLK6 [24,25] (*p* = 1.46e^−05^), BIN1 [26,27] (*p* = 4.11e^−05^), PSEN1 [21,22] (*p* = 0.00019), and LAMP1 [28,29] (*p* = 0.0005). 

### 3.2. Expression Quantitative Trait Loci

Our *p*-value distribution (Figure 5) looks to be well-normalized, which seems to indicate no major batch effects. In our initial results, we are able to successfully validate a number of previously identified LOAD-related genes (Table 3). The first column is the gene which contains the SNP identified in eQTL analysis. This column also contains a reference where the gene in question has been previously associated with LOAD through genome-wide association. Beta represents the effect size and direction. The third column is the Bonferroni corrected *p*-value. We observed strong associations in genes that are involved in the processing of the amyloid precursor protein, such as BACE2 and LRP2. Much to our surprise, only one of the 1,048,576 eQTL SNPs below the significance threshold (1e^−05^) overlaps genes from Table 2. NPC1, contained a SNP (rs5749088, *p* = 3.49e^−05^) predicted to alter gene expression this, of course, does not mean that these pathways are not being perturbed through germline mutation; however, we will continue to explore this observation in future studies with additional samples. 

### 3.3. Gene Set Enrichment Analysis

**Figure 6:**
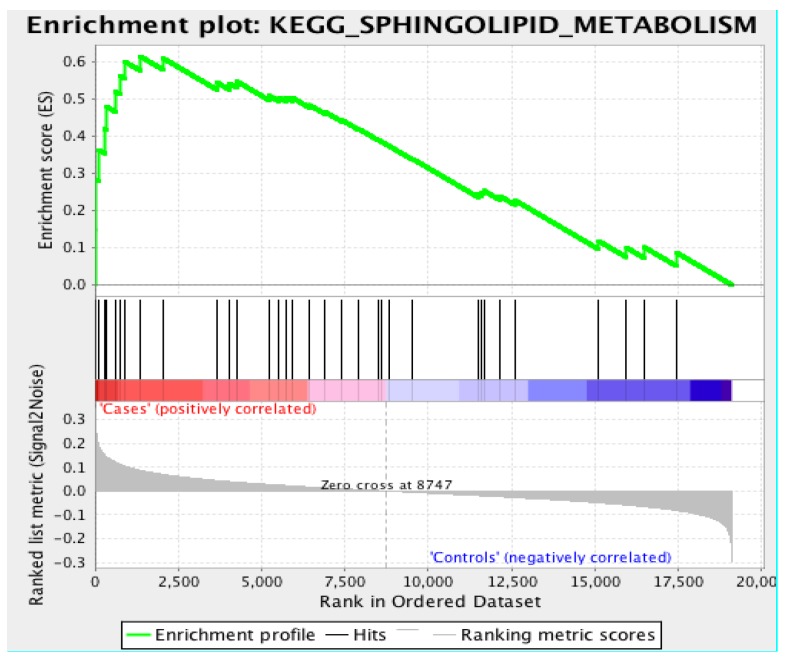
Enrichment plot for the sphingolipid metabolism pathway for ITG RNA-seq dataset based on case and control phenotypes and enriched using the KEGG biological pathway database.

**Figure 7:**
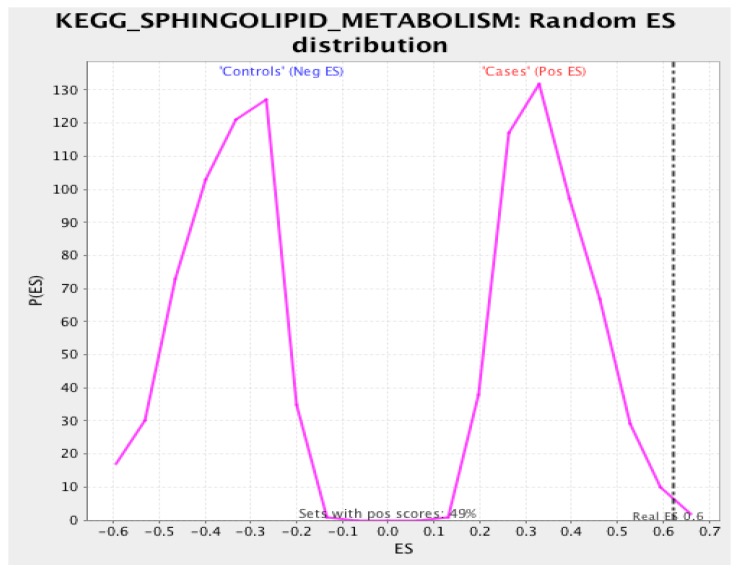
Null distribution of enrichment scores in the sphingolipid metabolism pathway in cases versus controls.

Using the KEGG pathway database, GSEA revealed the ‘sphingolipid metabolism’ pathway as the most significant in association with LOAD cases with an overall enrichment score (ES) of 0.617 and a normalized enrichment score (NES) of 1.731 (nominal *p*-value = 0.006, FDR = 0.476). Interestingly, we observed the ‘regulation of autophagy’ as the overall most significant association (nominal *p*-value = 0.002, FDR = 0.008) with an ES of −0.63 and an NES of −1.991, which suggests that genes related to the regulation of autophagy are enriched in controls, which is why the ES and NES are negative. Please see Table S2 for a list of all pathways and statistics. The NES is the ES divided by the mean of enrichment scores for all permutations of the dataset. This provides a standardized way in which phenotypes can be compared across a dataset. Figure 6 provides the enrichment plot for the sphingolipid metabolism pathway where controls are correlated with an overall decrease in enriched genes for this pathway. Unfortunately, our sample size does not allow for gene-level resolution, as the FDR is quite high at 0.476. However, we can clearly see a considerable difference (Figure 7) in the expression profiles of LOAD cases versus controls regarding the expression of genes involved in the metabolic processing of sphingolipids. 

## 4. Discussion

### 4.1. Biological Relevance

The objective of this study was two-fold: (1) to test the validity of our workflow and (2) to uncover biologically and statistically relevant pathways as they pertain to LOAD. This approach has allowed us to piece together some of the ways in which biological networks can be perturbed and/or are driven to dysfunction. Next, we will look more closely at the roles of the genes that we identified in our association analysis.

### 4.2. Sphingolipid Pathway

Glycolipid transfer protein (GLTP) is a highly conserved adhesion and transfer protein that selectively transports glycosphingolipids (GSLs) across the cell membrane to their respective microdomains or lipid rafts [36]. GSLs are essential for the proper functioning and maintenance of cells and are involved in a number of cellular processes, including the assembly of signaling molecules, membrane protein transport, and neurotransmission [37]. In a recent study, GLTP and MOBP were shown to be expressed together in the hippocampi of old mice. Interestingly, this study also uncovered evidence of differences in microglial-related neuroinflammation between male and female mice [38]. 

Niemann-Pick disease type C1 (NPC1), a late-endosomal, integral membrane protein, is a key component in cholesterol and sphingosine transport and homeostasis. Genetic defects on NPC1 cause a fatal accumulation of cholesterol in the late endosomes and lysosomes in lung, liver, and brain tissue [39,40,41]. NPC1 has been shown to be essential to the proper synthesis and maintenance of myelin in the CNS [42]. Ceramide synthase 2 (CERS2), also known as Longevity Assurance Homolog 2, is a key catalyzing agent in the synthesis of sphingomyelins, and its expression increases during times of myelination [43]. More pertinently, a considerable reduction (~25%) in CERS2 with aging has been observed in early and mid (I–IV), but not late (V–VI) Braak staging, suggesting that not only does demyelination precede tauopathy but also late stage Braak may be accelerated by non-genetic factors [38]. UGT8 (UDP glycosyltransferase 8) catalyzes the transfer of galactose to ceramide, which is necessary for the synthesis of galactocerebrosides, the primary components of the sphingolipids of the myelin membrane. The disruption of this pathway leads to demyelination in the PNS and CNS [44]; however, to our knowledge, there is currently no direct evidence to suggest that the expression of UGT8 is affected by the aging process.

It has been well known for some time that polyunsaturated fatty acids (PUFA) derived from omega-3 and other fatty acids play an important, protective role in neurodegeneration through their anti-oxidative effect and may even provide an additional means of prevention for neurodegeneration [45,46]. Fatty acid 2-hydroxylase (FA2H) catalyzes the synthesis of 2-hydroxysphingolipids and are necessary for the synthesis of ceramide. FA2H is essential to the proper functioning of the CNS throughout life [47]. Elongation of Very Long Chain Fatty Acids-Like 1 (ELOV1) is the first, rate-limiting reaction of the four major steps in the long-chain fatty acids elongation cycle. More saliently, it had been experimentally demonstrated that ELOVL1 activity is regulated by CERS2 [48]. Taken together, it seems reasonable to assume that a reduction of sphingomyelins and PUFAs could act to accelerate the demyelination process. 

### 4.3. Myelin Maintenance

NCAM1 or neural cell adhesion molecule 1 plays a critical role in synaptic bond stabilization and neuroplasticity. Loss of NCAM1 expression is associated with deficits in auditory and visual processing [49] and has been linked to LOAD. In aging rats, the NCAMs are significantly downregulated. ERMN, or juxtanodin, is an oligodendroglial protein that plays an important role in cytoskeletal rearrangements as well as in the maintenance of the myelin sheath [50]. It has yet to be demonstrated whether juxtanodin expression changes with age in the CNS; however, juxtanodin has been associated with age-related macular degeneration. 

Myelin-Associated Oligodendrocyte Basic Protein (MOBP) has been associated with progressive supranuclear palsy (PSP) [51], corticobasal degeneration (CBD), frontotemporal dementia (FTD) [52] and LOAD [51]. PSP, CBD, FTD, and LOAD all share tauopathy phenotypes. MOBP is also differentially expressed with aging [53]. Although there is much to be learned concerning the functions and pleiotropic effects of MOBP, it has been suggested that MOBP may act to compact and stabilize the myelin sheath [54]. Aspartoacylase (ASPA) codes for an enzyme that breaks down *N*-acetyl-l-aspartic acid (NAA), which is also essential to maintaining the myelin sheath [55]. Thinly myelinated neurons may be more susceptible to AD pathologies, such as amyloid deposits, NFTs, and neuroinflammation. 

ST18 is a member of the myelin transcription factor 1 (MTF1) family of neuronal-specific zinc finger proteins that regulates the transcription of myelin as well as apoptosis and inflammation [56]. SOX10 (SRY-Box 10) is a transcription factor that has been associated with the regulation of (de)myelination and age [57,58]. ST18 and SOX10 offer direct drivers for the transcriptional activity of myelination and therefore potential therapeutic targets. 

## 5. Conclusions

In summary, we have applied our co-expression modeling, functional and pathway perturbation analysis to the inferior temporal gyrus dataset provided by the MSBB Array Tissue Panel to test our workflow and better characterize the larger transcriptional landscape of late-onset Alzheimer’s disease with an emphasis on age-related physiopathological components. Association testing has revealed several key genes related to sphingolipid metabolism and myelin maintenance, as listed in Table 2. Specifically, CERS2 and UGT8 code for two enzymes that catalyze the synthesis of sphingomyelin, which are especially abundant in the myelin membrane. Finally, we used gene set enrichment analysis to independently validate the sphingolipid metabolic pathway as a statistically significant driver toward the AD phenotype. Perturbations to the autophagy-lysosomal pathway will require larger datasets to be confirmed, but it is tempting to speculate that both mechanisms are coordinated in aging and LOAD [59]. Do changes in sphingolipid metabolism lead to demyelination or a lack of remyelination as part of the aging process? Does demyelination or a lack of remyelination accelerate neuroinflammation and proceed tauopathy? Are these alterations present in other brain areas of LOAD patients? These are questions which we will continue to investigate in future studies.

## Figures and Tables

**Table 1:** Neuropathological Measures.

Trait	Description
CDR	Cognitive Dementia Rating no cognitive deficits (CDR = 0) questionable dementia (CDR = 0.5) mild dementia (CDR = 1.0) moderate dementia (CDR = 2.0) severe to terminal dementia (CDR = 3.0–5.0)
Braak	Quantitative assessment of neurofibrillary tangles (NTF) based anti-tau (AD2) staining
NP1	Neuropathology category
PLQ_Mn	Mean neocortical plaque density across 5 regions (# of plaques/mm^2^)
NTr_Sum	Sum of NFT density across 3 Brodmann areas
NPr_Sum	Sum of neuritic plaque density across 3 Brodmann areas

List of six neuropathology measures with brief description. Please see the website for more details: https://www.synapse.org/#!Synapse:syn5550382.

**Table 2:** Transcripts associated with myelin or sphingolipid.

GENE	P.WEIGHTED	FDR	COR.WEIGHTED	#OF TRAITS
GLTP	<4.88e^−09^	<2.22e^−16^	0.8465	3
NPC1	4.88e^−09^	2.22e^−16^	0.8395	3
CERS2	1.46e^−08^	2.22e^−12^	0.8307	3
ST18	3.41e^−08^	3.89e^−12^	0.8255	2
NCAM1	1.46e^−07^	1.02e^−11^	0.8151	3
ASPA	2.88e^−07^	1.54e^−11^	0.8103	2
ELOVL1	4.89e^−06^	3.53e^−11^	0.7944	3
ERMN	1.16e^−05^	6.48e^−11^	0.7945	3
MOBP	3.56e^−05^	1.60e^−10^	0.7851	2
FA2H	1.55e^−05^	3.22e^−10^	0.7774	1
UGT8	2.12e^−04^	3.54e^−09^	0.7691	2
SOX10	4.01e^−03^	3.44e^−07^	0.7622	3

List of gene transcripts sorted by P.WEIGHTED (Bonferroni corrected) that contained the term ‘myelin’ or ‘sphingolipid’ in their functional annotation.

**Table 3:** Gene Validation.

Gene	rsID	Beta	*p*-Value
GAS7 [30]	17339499	−1.129	8.60e^−10^
LRP2 [31]	2075252	−0.4257	3.15e^−07^
ABCA1 [32]	2230806	0.7890	1.20e^−05^
EPHA10 [33]	1212384	−0.3230	1.22e^−05^
ANK1 [34]	61063081	0.2079	1.30e^−05^
BACE2 [35]	2252576	0.4662	3.00e^−05^

List of previously identified, LOAD-related genes identified through eQTL analysis sorted by *p*-value.

## Supplementary Materials

The following are available online at http://www.mdpi.com/2079-7737/7/1/16/s1, Table S1: Association_Results.xlsx, Table S2: GSEA_Results.xlsx and Code S1: Process_Regions.docx.
