# Supplementary material for: Integrated Systems Approach Reveals Sphingolipid Metabolism Pathway Dysregulation in Association with Late-Onset Alzheimer’s Disease
Source: Biology (Basel). 2018 Feb 9;7(1):16. doi: 10.3390/biology7010016 (PMC5872042; doi:10.3390/biology7010016)
Supplement: Supplementary file 1 [file biology-07-00016-s001.pdf]

Table S1 Association Results

[Braak] Malamon, Kriete: Integrated Systems Approach, 2018

GeneName	p.Weighted	p.Weighted.adj	cor.Weighted	GO.term.1	GO.term.2
SLC44A1.2	3.55E-15	7.81E-08	0.819822422	choline transport	mitochondria
GLTP.1	5.77E-15	1.27E-07	0.816302445	lipid metabolic process	sphingolipid
MTMR10.1	6.39E-14	1.41E-06	0.797951424	cytosol	phosphatase
SOX8	2.37E-13	5.21E-06	0.787085514	transcription factor	neural crest
GPRC5B.1	4.19E-13	9.22E-06	0.782154937	integral membrane	G-protein
NCAM1.3	4.45E-13	9.79E-06	0.781624896	protein binding	myelin
SLC44A1.1	1.28E-12	2.82E-05	0.772132954	choline transport	mitochondria
FAM107B	1.56E-12	3.43E-05	0.77025677	mitochondria	mitochondria
UGT8.1	1.77E-12	3.89E-05	0.769099729	transferase	myelin
ERBB3.2	2.07E-12	4.55E-05	0.767631259	transcription factor	protein tyrosine kinase
MAN2A1	3.10E-12	6.82E-05	0.763759677	metabolic process	hydrolase activity
PLEKHH1.1	3.24E-12	7.13E-05	0.763337879	unknown	unknown
DOCK5.3	3.69E-12	8.12E-05	0.762087913	protein binding	cell adhesion
RNF130	3.69E-12	8.12E-05	0.762094156	membrane	metal ion binding
NPC1	6.50E-12	1.43E-04	0.756517114	cholesterol trafficking	sphingolipid
ERMN	7.22E-12	1.59E-04	0.755469786	actin binding	myelin
BOK	9.80E-12	2.16E-04	0.752383357	protein binding	apoptosis
CNTN2	1.54E-11	3.39E-04	0.747743281	unknown	unknown
ELOVL1	1.55E-11	3.41E-04	0.74764744	fatty acid	sphingolipid
DBNDD2	3.55E-11	7.81E-04	0.738878658	protein binding	neuron projection
LASS2	5.09E-11	1.12E-03	0.734954024	lipid metabolic process	myelin
C12orf34	7.57E-11	1.67E-03	0.730528911	unknown	unknown
LIPA	9.59E-11	2.11E-03	0.72786111	fatty acid	glycerolipid metabolic p
RDX.1	9.71E-11	2.14E-03	0.727716985	protein binding	RNA binding
PMP22	1.17E-10	2.57E-03	0.725555333	protein binding	myelin
HSPA2	1.19E-10	2.62E-03	0.725383098	protein binding	ATP binding
FRYL.1	1.55E-10	3.41E-03	0.722308176	transcription	neuron projection
MOBP.2	2.06E-10	4.53E-03	0.718995714	mitochondria	myelin
SPTLC2.3	2.25E-10	4.95E-03	0.717951958	transferase	sphingolipid
RTKN	2.87E-10	6.31E-03	0.715035254	protein binding	GTPase activity
DIP2B	3.89E-10	8.56E-03	0.711361409	catalytic activity	mental retardation
GAB1.1	5.12E-10	1.13E-02	0.707984476	cellular growth	apoptosis
SOX10	5.70E-10	1.25E-02	0.706653046	transcription	neuron projection
PLA2G16	6.32E-10	1.39E-02	0.705382229	protein binding	phospholipase
SGK3	6.72E-10	1.48E-02	0.704604225	calcium activity	apoptosis
ENPP2.1	6.96E-10	1.53E-02	0.704160319	lysophospholipase	oligodentrocyte
CERCAM	7.09E-10	1.56E-02	0.703928675	cell adhesion	blood-brain barrier
FEZ1	7.24E-10	1.59E-02	0.703677018	protein kinase C binding	gamma-tubulin binding
HIP1.2	7.24E-10	1.59E-02	0.703677601	protein binding	clathrin binding
LAMP2.1	8.11E-10	1.78E-02	0.702239825	protein binding	lysosome
ST18	1.34E-09	2.95E-02	0.695833169	transcription factor	myelin
EDIL3.1	1.35E-09	2.97E-02	0.695703049	protein binding	calcium
CPNE2	1.51E-09	3.32E-02	0.694261029	protein binding	calcium
FAM107B.1	1.88E-09	4.14E-02	0.691375155	mitochondria	mitochondria
SEMA4D	1.89E-09	4.16E-02	0.691277199	recptor activity	transcription regulation
GPRC5B.2	1.95E-09	4.29E-02	0.690870146	integral membrane	G-protein
ASPA.1	2.21E-09	4.86E-02	0.689185743	hydrolase	myelin

[NP1]

GeneName	p.Weighted	p.Weighted.adj	cor.Weighted	GO.term.1	GO.term.2
CXXC5	1.75E-10	3.85E-03	0.720928752	DNA binding	zinc ion binding
ANKIB1.1	1.11E-09	2.44E-02	0.69823082	transferase	metal ion binding
PTPLAD1.3	1.73E-09	3.81E-02	0.692425385	fatty acid	sphingolipid
UBE2R2	2.02E-09	4.44E-02	0.690414044	transferase	ATP binding
PTPLAD1.1	2.15E-09	4.73E-02	0.689563836	fatty acid	sphingolipid

[NTr_Sum]

GeneName	p.Weighted	p.Weighted.adj	cor.Weightec	GO.term.1	GO.term.2
GLTP.1	5.27E-13	1.16E-05	0.78013483	lipid metabolic process	sphingolipid
SOX8	2.14E-12	4.71E-05	0.76731834	transcription factor	neural crest
MTMR10.1	4.61E-12	1.01E-04	0.75991459	cytosol	phosphatase
FAM107B	4.84E-12	1.06E-04	0.7594361	mitochondria	mitochondria
NCAM1.3	5.92E-12	1.30E-04	0.75745578	protein binding	myelin
SLC44A1.1	6.78E-12	1.49E-04	0.75609301	choline transport	mitochondria
LASS2	9.86E-12	2.17E-04	0.75232823	lipid metabolic process	myelin
MAN2A1	1.28E-11	2.82E-04	0.74966369	metabolic process	hydrolase activity
NPC1	2.06E-11	4.53E-04	0.74471588	cholesterol trafficking	sphingolipid
RNF130	2.72E-11	5.98E-04	0.74173882	membrane	metal ion binding
SLC44A1.2	2.73E-11	6.01E-04	0.74169347	choline transport	mitochondria
GPRC5B.1	2.80E-11	6.16E-04	0.74144289	integral membrane	G-protein
FRYL.1	2.87E-11	6.31E-04	0.74118365	transcription	neuron projection
CPNE2	4.62E-11	1.02E-03	0.73600973	protein binding	calcium
CNTN2	5.45E-11	1.20E-03	0.73418783	unknown	unknown
BOK	5.65E-11	1.24E-03	0.73379193	protein binding	apoptosis
PLEKHH1.1	7.11E-11	1.56E-03	0.73123705	unknown	unknown
ERBB3.2	7.99E-11	1.76E-03	0.72992277	transcription factor	protein tyrosine kinase
ELOVL1	1.34E-10	2.95E-03	0.72401055	fatty acid	sphingolipid
LIPA	1.77E-10	3.89E-03	0.72075713	fatty acid	glycerolipid metabolic process
PLA2G16	2.18E-10	4.80E-03	0.71835092	protein binding	phospholipase
DOCK5.3	2.35E-10	5.17E-03	0.71743296	protein binding	cell adhesion
C12orf34	2.61E-10	5.74E-03	0.71620658	unknown	unknown
SGK3	3.28E-10	7.22E-03	0.71345487	calcium activity	apoptosis
TMEM87A	4.12E-10	9.06E-03	0.7106723	membrane	Golgi
SOX10	4.24E-10	9.33E-03	0.71030528	transcription	neuron projection
PMP22	5.04E-10	1.11E-02	0.70818804	protein binding	myelin
ERMN	5.55E-10	1.22E-02	0.70699318	actin binding	myelin
RDX.1	5.88E-10	1.29E-02	0.7062694	protein binding	RNA binding
DBNDD2	6.89E-10	1.52E-02	0.70429851	protein binding	neuron projection
SEMA4D	1.86E-09	4.09E-02	0.69151446	recptor activity	transcription regulation
HSPA2	1.93E-09	4.25E-02	0.69099753	protein binding	ATP binding
PREX1.1	1.96E-09	4.31E-02	0.69076784	protein binding	GTPase activity
CNTNAP4	1.97E-09	4.33E-02	0.69070537	protein binding	synapse
PPAP2C	2.18E-09	4.80E-02	0.68935082	phosphatase	receptor activity

[PLQ_Mn]

GeneName	p.Weighted	p.Weighted.adj	cor.Weighted	Go.term.1	Go.Term1
GLTP.1	0	0	0.846564448	lipid metabolic process	sphingolipid
ERBB3.2	0	0	0.846991467	transcription factor	protein tyrosine kinase
MAN2A1	0	0	0.843113087	metabolic process	hydrolase activity
CNTN2	0	0	0.853491977	unknown	unknown
NPC1	2.22E-16	4.88E-09	0.839525155	cholesterol trafficking	sphingolipid
LASS2	6.66E-16	1.4652E-08	0.830793058	lipid metabolic process	myelin
PLEKHH1.1	8.88E-16	1.9536E-08	0.830052561	unknown	unknown
ST18	1.55E-15	3.41E-08	0.825521306	transcription factor	myelin
SLC44A1.1	2.00E-15	4.4E-08	0.823833068	choline transport	mitochondria
SEMA4D	3.77E-15	8.294E-08	0.819395067	recptor activity	transcription regulation
SOX8	4.22E-15	9.284E-08	0.818620608	transcription factor	neural crest
DBNDD2	6.22E-15	1.3684E-07	0.815971101	protein binding	neuron projection
NCAM1.3	6.66E-15	1.4652E-07	0.815110715	protein binding	myelin
FAM107B	7.99E-15	1.7578E-07	0.813825438	mitochondria	mitochondria
TMEM87A	9.33E-15	2.0526E-07	0.812720067	membrane	Golgi
FRYL.1	1.02E-14	2.244E-07	0.812241436	transcription	neuron projection
ASPA.1	1.31E-14	2.882E-07	0.810300798	hydrolase	myelin
USP54	2.09E-14	4.598E-07	0.806711921	protein binding	hydrolase activity
CNTNAP4	2.40E-14	5.28E-07	0.805737146	protein binding	synapse
RHOU	2.58E-14	5.676E-07	0.805125296	protein binding	GTPase activity
DOCK5.3	2.93E-14	6.446E-07	0.804107659	protein binding	cell adhesion
TTYH2	3.51E-14	7.722E-07	0.802731441	calcium activity	chloride channel
ELOVL1	4.06E-14	8.932E-07	0.801585002	fatty acid	sphingolipid
C12orf34	5.24E-14	1.1528E-06	0.799535136	unknown	unknown
PLA2G16	6.66E-14	1.4652E-06	0.79764294	protein binding	phospholipase
JAM3	6.71E-14	1.4762E-06	0.797582997	protein binding	junction
MTMR10.1	6.71E-14	1.4762E-06	0.797570176	cytosol	phosphatase
CPNE2	7.31E-14	1.6082E-06	0.796881927	protein binding	calcium
SLC44A1.2	7.99E-14	1.7578E-06	0.796167359	choline transport	mitochondria
ERMN	9.73E-14	2.1406E-06	0.79456505	actin binding	myelin
FNBP1	1.22E-13	2.684E-06	0.792699497	protein binding	endocytosis
SGK3	1.36E-13	2.992E-06	0.791771826	calcium activity	apoptosis
PMP22	1.98E-13	4.356E-06	0.788646384	protein binding	myelin (only PNS)
PDE1C	2.55E-13	5.61E-06	0.786475357	hydrolase	calmodulin binding
ELOVL1.1	2.65E-13	5.83E-06	0.78612844	fatty acid	sphingolipid
MOBP.2	2.97E-13	6.534E-06	0.785162617	mitochondria	myelin
HSPA2	3.07E-13	6.754E-06	0.784863219	protein binding	ATP binding

PPP1R14A	3.98E-13	8.756E-06	0.782621026	cytoplasm	phosphorylatin
RDX.1	4.28E-13	9.416E-06	0.781969058	protein binding	RNA binding
BOK	4.46E-13	9.812E-06	0.781621094	protein binding	apoptosis
HIP1.2	4.52E-13	9.944E-06	0.78149265	protein binding	clathrin binding
KLK6	6.67E-13	1.4674E-05	0.778034494	hydrolase	amyloid
FA2H	7.09E-13	1.5598E-05	0.777494699	fatty acid	sphingolipid
GPRC5B.1	1.26E-12	2.772E-05	0.772278384	integral membrane	G-protein
FAM124A	1.32E-12	2.904E-05	0.771855521	protein binding	protein binding
C11orf9	1.52E-12	3.344E-05	0.77053349	transcription factor	myelin
PREX1.1	1.75E-12	3.85E-05	0.769202252	protein binding	GTPase activity
RTKN	1.91E-12	4.202E-05	0.768406245	protein binding	GTPase activity
ABCA8	1.91E-12	4.202E-05	0.768366551	ATP binding	myelin
UGT8.1	1.99E-12	4.378E-05	0.767974539	transferase	myelin
ARHGEF37	2.36E-12	5.192E-05	0.766397112	cytoplasm	GTPase activity
ZNF536	2.74E-12	6.028E-05	0.764944019	transcription	RNA polymerase II
DIP2B	3.06E-12	6.732E-05	0.76389877	catalytic activity	mental retardation
TBC1D12	3.53E-12	7.766E-05	0.762509428	GTPase activity	autophagosome
DYNC1LI2.:	4.24E-12	9.328E-05	0.760737225	microtubule	dynein heavy chanin binding
PHLPP1	4.32E-12	9.504E-05	0.76054572	cell signaling	apoptosis
GAL3ST1	4.37E-12	9.614E-05	0.760448148	glycolipids	Golgi
C21orf91	4.75E-12	0.0001045	0.759629762	neuron differentiation	dendritic spine
ASPA	5.02E-12	0.00011044	0.75907948	hydrolase	myelin
EDIL3.1	8.21E-12	0.00018062	0.754179845	protein binding	calcium
ALCAM.1	8.41E-12	0.00018502	0.753938213	recptor activity	immunoglobulin
PSEN1	8.88E-12	0.00019536	0.753382682	cell junction	synapse
PCSK6	9.18E-12	0.00020196	0.753050755	proteolysis	serine-type endopeptidase
TMCC3	9.76E-12	0.00021472	0.752422812	membrane	membrane
SLC5A11	9.87E-12	0.00021714	0.752310887	transporter activity	sodium transport
FOLH1B	9.94E-12	0.00021868	0.752243925	proteolysis	metabolic process
RNF130	1.01E-11	0.0002222	0.75212396	membrane	metal ion binding
LIPA	1.16E-11	0.0002552	0.750692357	fatty acid	glycerolipid metabolic process
EVI2A	1.28E-11	0.0002816	0.74964843	membrane	membrane
CERCAM	1.34E-11	0.0002948	0.749165436	cell adhesion	blood-brain barrier
TMEM144	1.47E-11	0.0003234	0.748247676	membrane	carbohydrate transport
VEZF1.1	2.13E-11	0.0004686	0.744325285	metal ion binding	nucleic acid binding
CAPN3	2.17E-11	0.0004774	0.744136509	proteolysis	hydrolase activity
C14orf139	2.46E-11	0.0005412	0.742803694	protein binding	nuclear envelope
ENPP2	2.55E-11	0.000561	0.742445643	lysophospholipase	oligodentrocyte
LAMP1.1	2.60E-11	0.000572	0.742242959	membrane	lysosome

TJP2	2.63E-11	0.0005786	0.742124888	tight junction	metal ion binding
GOLGA7	2.63E-11	0.0005786	0.742124151	Golgi stack	protein targeting
FOXN2.1	3.25E-11	0.000715	0.739846446	DNA binding	transcription regulation
TMEM125	3.42E-11	0.0007524	0.739278878	membrane	membrane
LITAF	4.07E-11	0.0008954	0.737404219	DNA binding	transcription regulation
DOCK10.1	4.16E-11	0.0009152	0.737155531	GTPase activity	guanosine nucleotide exchange factors
SLC31A2	4.30E-11	0.000946	0.736810155	membrane	copper ion transport
ARHGAP23	7.15E-11	0.001573	0.731177296	GTPase activity	exosome
IPO13	7.63E-11	0.0016786	0.730442684	GTPase activity	nuclear membrane
CREB5.1	7.72E-11	0.0016984	0.730313266	DNA binding	cAMP
NASP.1	8.39E-11	0.0018458	0.729373551	germline	germline
PIP4K2A	9.21E-11	0.0020262	0.728317076	protein binding	signaling
FOLH1	9.66E-11	0.0021252	0.727773401	catalytic activity	folic acid
ARAP2	9.75E-11	0.002145	0.727676843	GTPase activity	signaling
ABCA2.1	9.75E-11	0.002145	0.727671276	lipid metabolic process	lysosome
PLEKHH1	1.04E-10	0.002288	0.726964535	unknown	unknown
FGFR2	1.30E-10	0.00286	0.724337503	cell division	cell growth
KIF13B	1.43E-10	0.003146	0.723285957	protein kinase	microtubule
HNRNPA2B	1.45E-10	0.00319	0.723127535	membrane	transcription regulation
TMEM206.1	1.51E-10	0.003322	0.72264808	membrane	cell surface
FAM124A.1	1.58E-10	0.003476	0.722114545	protein binding	protein binding
CLMN	1.61E-10	0.003542	0.721903085	actin binding	membrane
CDKN1C	1.76E-10	0.003872	0.72085838	mitosis	apoptosis
CARNS1	1.79E-10	0.003938	0.720647703	ATPase	carnosine
ENPP2.1	1.84E-10	0.004048	0.720349303	lysophospholipase	oligodentrocyte
TMTC4	2.01E-10	0.004422	0.719270193	membrane	membrane
GAB1.3	2.15E-10	0.00473	0.718473291	cell growth	apoptosis
CDK18	2.21E-10	0.004862	0.718149219	ATPase	protein kinase activity
ACER3.1	2.26E-10	0.004972	0.717922256	metabolic process	sphingolipid
SOX10	2.33E-10	0.005126	0.7175199	transcription	neuron projection
PLEKHG3	2.54E-10	0.005588	0.7165057	Rho guanyl-nucleotide exchange factor activity	
S1PR5	2.83E-10	0.006226	0.71520438	G-coupled protein	sphingolipid
VAMP3	2.89E-10	0.006358	0.714976548	vesicle fusion	SNARE
NIPAL3.3	3.05E-10	0.00671	0.714338407	magnesium ion transport	
SLC48A1	3.22E-10	0.007084	0.713661719	endosome	lysosome
COL4A5	3.52E-10	0.007744	0.71259064	collagen	collagen
TF	3.67E-10	0.008074	0.712092117	protein binding	ferrous binding
RASGRP3	3.77E-10	0.008294	0.711759669	GTPase activity	calcium
ANLN	3.84E-10	0.008448	0.711534724	actin binding	cadherin binding

QKI.4	4.61E-10	0.010142	0.709289225	RNA binding	RNA binding
TJAP1	5.01E-10	0.011022	0.70825505	tight junction	tight junction
MOG.2	5.38E-10	0.011836	0.707387484	transferase	transferase
PRR18	5.76E-10	0.012672	0.706541549	unknown	unknown
MAP7.1	6.10E-10	0.01342	0.705822279	cytoskeleton	microtubule
MOG	6.66E-10	0.014652	0.704723651	cell adhesion	myelin
ANKRD13A	6.70E-10	0.01474	0.70464865	cytoplasm	cytoplasm
PDE8A.1	7.59E-10	0.016698	0.703080326	hydrolase	cAMP
TJAP1.1	1.21E-09	0.02662	0.697153897	tight junction	tight junction
PAQR4	1.27E-09	0.02794	0.696528892	membrane	receptor activity
MTUS1.2	1.29E-09	0.02838	0.696244119	mitochondria	microtubule
PRIMA1	1.33E-09	0.02926	0.695861159	acetylcholinesterase	synapse
PPAP2C	1.54E-09	0.03388	0.693936347	phosphatase	receptor activity
GSN	1.60E-09	0.0352	0.693471169	actin binding	calcium
FOLH1.1	1.66E-09	0.03652	0.692991698	catalytic activity	folic acid
SLC44A1.3	1.66E-09	0.03652	0.692984157	choline transport	mitochondria
ZNF24.2	1.71E-09	0.03762	0.692559042	DNA binding	myelin
FRMD4B	1.72E-09	0.03784	0.692549233	cytoskeleton	cytoskeleton
SPTLC2.3	2.02E-09	0.04444	0.690421442	transferase	sphingolipid
TMEM98	2.03E-09	0.04466	0.690311453	membrane	membrane

Table S2 GSEA Results

Pathway	Size	ES	NES	NOM p-value	FDR
KEGG_SPHINGOLIPID_METABOLISM	34	0.617	1.731	0.006	0.479
KEGG_NITROGEN_METABOLISM	23	0.477	1.475	0.027	1
KEGG_BETA_ALANINE_METABOLISM	22	0.468	1.476	0.032	1
KEGG_HISTIDINE_METABOLISM	28	0.484	1.5	0.044	1
KEGG_DNA_REPLICATION	36	0.404	1.437	0.06	1
KEGG_PPAR_SIGNALING_PATHWAY	68	0.347	1.338	0.062	1
KEGG_GLUTATHIONE_METABOLISM	47	0.422	1.323	0.115	1
KEGG_PROPANOATE_METABOLISM	31	0.429	1.295	0.123	1
KEGG_INSULIN_SIGNALING_PATHWAY	134	0.33	1.283	0.125	1
KEGG_LEUKOCYTE_TRANSENDOTHELIAL_MIGRATION	110	0.331	1.28	0.126	1
KEGG_TGF_BETA_SIGNALING_PATHWAY	82	0.35	1.265	0.139	1
KEGG_SMALL_CELL_LUNG_CANCER	84	0.284	1.195	0.162	1
KEGG_GLYCEROLIPID_METABOLISM	46	0.335	1.21	0.164	1
KEGG_PRION_DISEASES	35	0.479	1.279	0.181	1
KEGG_N_GLYCAN_BIOSYNTHESIS	44	0.363	1.236	0.203	1
KEGG_VASCULAR_SMOOTH_MUSCLE_CONTRACTION	109	0.281	1.154	0.207	1
KEGG_BIOSYNTHESIS_OF_UNSATURATED_FATTY_ACIDS	20	0.382	1.186	0.223	1
KEGG_REGULATION_OF_ACTIN_CYTOSKELETON	207	0.29	1.156	0.224	1
KEGG_CHRONIC_MYELOID_LEUKEMIA	72	0.347	1.177	0.227	1
KEGG_FOCAL_ADHESION	197	0.267	1.139	0.23	1
KEGG_PATHWAYS_IN_CANCER	323	0.246	1.119	0.239	1
KEGG_LEISHMANIA_INFECTION	66	0.438	1.248	0.242	1
KEGG_FATTY_ACID_METABOLISM	39	0.358	1.16	0.243	1
KEGG_CYTOSOLIC_DNA_SENSING_PATHWAY	53	-0.449	-1.356	0.144	1
KEGG_GLYCOSAMINOGLYCAN_DEGRADATION	21	-0.433	-1.365	0.103	1
KEGG_RIBOFLAVIN_METABOLISM	16	-0.493	-1.381	0.105	1
KEGG_GALACTOSE_METABOLISM	26	-0.455	-1.538	0.036	1
KEGG_REGULATION_OF_AUTOPHAGY	32	-0.629	-1.991	0.002	0.008

```
library(WGCNA)
library(flashClust)

### Clear all objects. R doesn’t do memory management
rm(list=ls())
brain.region="ITG"

options(stringsAsFactors = FALSE)
enableWGCNAThreads()
home.dir = paste("/home/jmalamon/",brain.region,sep="")

home.dir="/Users/johnslaptop/Documents/GRAD/THESIS/MSBB/all"
datExpr=read.csv(paste(home.dir, "/AMP-
AD_MSBB_MSSM_IlluminaHiSeq2500_raw_counts_September_2016.txt",sep=""),sep
="\t");

### Load Expression data
datExpr=read.csv(paste(home.dir, "/AMP-
AD_MSBB_MSSM_AffymetrixU133AB_Inferior",sep=""),sep="\t");
### Prepare for processing
gene.names<-subset(datExpr, select = c(3,4))
datExpr[,1] <- NULL
datExpr <- log2(datExpr+1)
datExpr = as.data.frame(t(datExpr))
colnames(datExpr)<-as.character(gene.names[[1]])

### Methylation expression data
datExpr=read.csv(paste("/home/jmalamon/ROSMAP/ROSMAP_arrayMethylation_imput
ed.tsv",sep=""),sep="\t");
### Prepare for processing
gene.names<-subset(datExpr, select = c(1))
datExpr[,1] <- NULL
datExpr <- log2(datExpr+1)
datExpr = as.data.frame(t(datExpr))
colnames(datExpr)<-as.character(gene.names[[1]])

load(file="/home/jmalamon/ROSMAP/net_meth.RData")

### Calculate SFT
power=14
k1=softConnectivity(datExpr,corFnc="cor",corOptions="use='p'",type="signed",power=
power)
kCut = 50000
kRank = rank(-k1)
vardataOne=apply(datExpr,2,var)
vardataOne=apply(meth.df,2,var)

restk = kRank <= kCut & vardataOne>0
sum(restk)
```

```
### Calculate matrices
ADJ=adjacency(datExpr=meth.df[,restk],power=power);
dissTOM=TOMdist(ADJ)
hierTOM = hclust(as.dist(dissTOM),method="average");


### NETWORK CONTRUCTION
power = 12
deepSplit = 2
minModuleSize = 20
networkType = "signed"
TOMType = "signed"
TOMDenom = "mean"
reassignThreshold = 0
mergeCutHeight = 0.25

net<-blockwiseModules(
replace = TRUE,
datExpr = datExpr[,restk],
corType="bicor",
maxBlockSize = 40000,
power = power,
networkType = networkType,
TOMType = TOMType,
TOMDenom = TOMDenom,
deepSplit = deepSplit,
mergeCutHeight = mergeCutHeight,
reassignThreshold = reassignThreshold,
numericLabels = TRUE,
checkMissingData = FALSE,
pamStage = TRUE,
quickCor = 0, verbose = 5 )


### Branch cutting
cutHeight=0.997
minModuleSize = 15;
dynamicMods = cutreeDynamic(dendro=hierTOM, distM=dissTOM,
deepSplit=3,pamRespectsDendro=TRUE, pamStage=TRUE,
minClusterSize=minModuleSize,
cutHeight=cutHeight,method="hybrid",minAbsGap=0.001,respectSmallClusters=TRUE,
useMedoids=TRUE);
dynamicColors = labels2colors(dynamicMods)
module_colors= setdiff(unique(dynamicColors), "grey")
table(dynamicColors)

### Merge modules
```

```
merge = mergeCloseModules(datExpr[,restk], dynamicColors, cutHeight = 0.17, verbose
= 5)
mergedColors = merge$colors;
#mergedMEs = merge$newMEs;
table(mergedColors)

### YOU HAVE TO LOOK AT THE DENDROGRAM TO APPLY BRANCH
CUTTING PROPERLY
#modColors=mergedColors
modColors=dynamicColors
### Plot Dendrogram with modules
par(mfrow=c(1,1))
pdf(file="./Dendrogram_01.pdf", wi=20, h=15)
plotDendroAndColors(hierTOM,modColors,c("Module membership", "2 blocks"),main =
"Single block gene dendrogram and module colors",dendroLabels = FALSE, hang =
0.03,addGuide = TRUE, guideHang = 0.05,abHeight=cutHeight)
dev.off()

### Now load the clinical data
excel.file="/home/jmalamon/ITG/MSBB_MSSM_ITG_CLINICAL.csv"
clinical <- read.csv(file=excel.file,header=TRUE, sep=",")

### Plot heatmap of clinical correlations
library(corrplot)
par(mfrow=c(1,1))
pdf(file="./clinical_corr_01.pdf", wi=20, h=15)
clinical.matrix <- select(clinical$CDR, clinical$Braak, clinical$NP1, clinical$PLQ_0n,
clinical$NPrSu0, clinical$NTrSu0)
corr.matrix<- round(cor(clinical[,7:12]), 2)
corrplot(corr.matrix, method = "color",addCoef.col = "black"
)
dev.off()

### write all gene names for each module
for (color in mergedColors){
        module=gene.names[which(mergedColors==color),1]
        input.file.name<-paste("./module_",color,".txt",sep="")
        write.table(module,input.file.name, sep="\t", row.names=FALSE,
        col.names=FALSE,quote=FALSE)
        #input.table <-
        read.csv(input.file.name,sep="\n",header=FALSE,comment.char="",stringsAsFac
        tors=FALSE)
}

### Calculate Principle Components
PCs = moduleEigengenes(datExpr[,restk],mergedColors)$eigengenes

### Network FPR screening (Noise or FPR rate)
NS1=networkScreening(y=clinical$CDR, datME=PCs, datExpr=datExpr[,restk],
```

```
oddPower=3, blockSize=1000, minimumSampleSize=4,
addMEy=TRUE, removeDiag=FALSE, weightESy=0.5)

NS2=networkScreening(y=clinical$Braak, datME=PCs, datExpr=datExpr[,restk],
oddPower=3, blockSize=1000, minimumSampleSize=4,
addMEy=TRUE, removeDiag=FALSE, weightESy=0.5)

NS3=networkScreening(y=clinical$PLQ_0n, datME=PCs, datExpr=datExpr[,restk],
oddPower=3, blockSize=1000, minimumSampleSize=4,
addMEy=TRUE, removeDiag=FALSE, weightESy=0.5)

NS4=networkScreening(y=clinical$NPrSu0, datME=PCs, datExpr=datExpr[,restk],
oddPower=3, blockSize=1000, minimumSampleSize=4,
addMEy=TRUE, removeDiag=FALSE, weightESy=0.5)

NS5=networkScreening(y=clinical$NTrSu0, datME=PCs, datExpr=datExpr[,restk],
oddPower=3, blockSize=1000, minimumSampleSize=4,
addMEy=TRUE, removeDiag=FALSE, weightESy=0.5)

NS6=networkScreening(y=clinical$NP1, datME=PCs, datExpr=datExpr[,restk],
oddPower=3, blockSize=1000, minimumSampleSize=4,
addMEy=TRUE, removeDiag=FALSE, weightESy=0.5)

# Write the data frame into an excel file
GeneResultsNetworkScreening1=data.frame(GeneName=row.names(NS1), NS1)
write.table(GeneResultsNetworkScreening1,
file="GeneResultsNetworkScreening_CDR.csv", row.names=F,sep=",")

GeneResultsNetworkScreening2=data.frame(GeneName=row.names(NS2), NS2)
write.table(GeneResultsNetworkScreening2,
file="GeneResultsNetworkScreening_Braak.csv", row.names=F,sep=",")

GeneResultsNetworkScreening3=data.frame(GeneName=row.names(NS3), NS3)
write.table(GeneResultsNetworkScreening3,
file="GeneResultsNetworkScreening_PLQ_Mn.csv", row.names=F,sep=",")

GeneResultsNetworkScreening4=data.frame(GeneName=row.names(NS4), NS4)
write.table(GeneResultsNetworkScreening4,
file="GeneResultsNetworkScreening_NPrSum.csv", row.names=F,sep=",")

GeneResultsNetworkScreening5=data.frame(GeneName=row.names(NS5), NS5)
write.table(GeneResultsNetworkScreening5,
file="GeneResultsNetworkScreening_NTrSum.csv", row.names=F,sep=",")

GeneResultsNetworkScreening6=data.frame(GeneName=row.names(NS6), NS6)
write.table(GeneResultsNetworkScreening6,
file="GeneResultsNetworkScreening_NP1.csv", row.names=F,sep=",")

### Calculate intramodular connectivity for top genes
```

```
ADJ1=abs(cor(datExpr[,restk],use="p"))^6
Alldegrees=intramodularConnectivity(ADJ1,mergedColors)
Alldegrees.sorted <- Alldegrees[order(Alldegrees$kTotal) , ]
head(Alldegrees)

# Write the data frame into a file
write.table(Alldegrees.sorted, file="Alldegrees_01.csv",row.names=TRUE,sep=",")

par(mfrow=c(1,2))
pdf(file="./Alldegrees_01.pdf", wi=20, h=15)
myhist <-
hist(Alldegrees[,1],prob=FALSE,col="blue",xlim=c(0,900),labels=TRUE,main="Networ
k Connectivity (degrees) by Transcript",xlab="degrees")
dens <- density(Alldegrees.sorted[,1])
axis(side=1, at=seq(0,20,1))
#lines (density(Alldegrees.sorted[,1]), col="red")
abline(v=76, col="red")
dev.off()

### Run DAVID analysis and get GO annotation
LLIDs = list()
n=20000
for (i in 1:n) {
tempID=gene.names[match(colnames(datExpr)[n],gene.names[,1]),]
  n=n+1;
LLIDs[[i]]=tempID[[2]]
}
allLLIDs<-unlist(LLIDs, recursive = TRUE)

library("org.Hs.eg.db")
GOenr = GOenrichmentAnalysis(mergedColors, allLLIDs, organism = "human", nBestP
= 20, evidence = "all",getTermDetails = TRUE, verbose = 5, indent = 0 );

tab = GOenr$bestPTerms[[4]]$enrichment
### Write to Excel
write.table(tab, file = "GOEnrichmentTable_ITG_all.csv", sep = ",", quote = TRUE,
row.names = FALSE)
keepCols = c(1,2,5,6,7,11,12,13);
screenTab = tab[, keepCols];

### PCA on clinical data
require(FactoMineR)
library("factoextra")
require(ggplot2)
library("missMDA")
library("corrplot")

clinical.df=read.csv("/Users/johnslaptop/Documents/GRAD/DISSERTATION/MSBB/cli
nical/AMP-AD_MSBB_MSSM_covariates_mRNA_AffymetrixU133AB.csv");
```

```
row.names(clinical.df)<-clinical.df[,1]
clinical.df[,1]=NULL
clinical.df[,3:4]=NULL

res.pca = PCA(clinical.df[,1:11], scale.unit=TRUE, ncp=5, graph=T)

fviz_pca_var(res.pca, col.var="contrib") + scale_color_gradient2(low="white",
mid="yellow", high="red", midpoint=0.50)+theme_minimal()
```
